# Telemedicine-delivered cognitive-behavioral therapy for insomnia in alcohol use disorder (AUD): study protocol for a randomized controlled trial

**DOI:** 10.1186/s13063-021-05898-y

**Published:** 2022-01-20

**Authors:** J. Todd Arnedt, M. Elizabeth Cardoni, Deirdre A. Conroy, Mandilyn Graham, Sajni Amin, Kipling M. Bohnert, Andrew D. Krystal, Mark A. Ilgen

**Affiliations:** 1 Sleep and Circadian Research Laboratory, Department of Psychiatry, Michigan Medicine, University of Michigan, 4250 Plymouth Road, Ann Arbor, MI 48109-2700 USA; 2 Department of Epidemiology and Biostatistics, Michigan State University, East Lansing, USA; 3 UCSF Weill Institute for Neurosciences, University of California San Francisco, San Francisco, USA; 4 University of Michigan Addiction Treatment Services, Department of Psychiatry, Michigan Medicine, University of Michigan, Ann Arbor, USA; 5 VA Center for Clinical Management Research (CCMR) Department of Veterans Affairs Healthcare System, Michigan Ann Arbor, USA

**Keywords:** Alcohol use disorder, Insomnia, Non-medication treatment, Relapse

## Abstract

**Background:**

Alcohol use disorder (AUD) is a leading preventable cause of morbidity and mortality, but relapse rates are high even with available treatments. Insomnia is a robust predictor of relapse and pilot studies have shown that CBT for insomnia improves insomnia and daytime functioning in adults with AUD and insomnia. The impact of CBT for insomnia on relapse, however, is unclear. This trial will compare telemedicine-delivered CBT for insomnia (CBT-TM) with sleep hygiene education (SHE-TM) on improving insomnia/sleep, daytime symptom, and drinking outcomes in treatment-seeking AUD adults with insomnia. The study will also determine the effects of treatment on sleep mechanisms and their association with clinical outcomes.

**Methods:**

This is a single-site randomized controlled trial with planned enrollment of 150 adults meeting criteria for both AUD and chronic insomnia. Eligible participants will be randomized 1:1 to 6 sessions of telemedicine-delivered Cognitive Behavioral Therapy for Insomnia (CBT-TM) or Sleep Hygiene Education (SHE-TM) with clinical assessments conducted at pre-treatment, post- treatment, and at 3, 6, and 12 months post-treatment. Overnight polysomnography will be conducted before and after treatment. Primary clinical outcomes will include post-treatment scores on the Insomnia Severity Index and the General Fatigue subscale of the Multidisciplinary Fatigue Inventory, and the percent of days abstinent (PDA) on the interview-administered Time Line Follow Back. EEG delta activity, derived from overnight polysomnography, will be the primary endpoint to assess the sleep homeostasis mechanism.

**Discussion:**

This adequately powered randomized controlled trial will provide clinically relevant information about whether targeting insomnia is effective for improving treatment outcomes among treatment-seeking adults with AUD. Additionally, the study will offer new scientific insights on the impact of an evidence-based non-medication treatment for insomnia on a candidate mechanism of sleep dysfunction in this population - sleep homeostasis.

**Trial registration:**

CClinicalTrials.gov NCT # 04457674. Registered on 07 July 2020.

## Background

Alcohol use disorder (AUD) is a leading preventable cause of morbidity and mortality in the United States (US), with 12-month and lifetime prevalence estimates of 13.9% and 29.1%, respectively [1]. It accounts for approximately 88,000 deaths annually [1–4] and ranks as a leading cause of disability burden worldwide [5–7], contributing to multiple diseases [8–10], and to substantial medical, legal, and social problems for afflicted individuals and their families. Existing AUD treatments are effective; however, relapse rates remain high following treatment. Novel approaches that target predictors of relapse are urgently needed to enhance existing AUD treatments, improve clinical outcomes, and reduce the overall burden of AUD.

Insomnia is one especially promising treatment target for individuals with AUD. It is more prevalent in adults with AUD than in the general population, with up to 74% among those actively drinking, 69% during early recovery, and 50% after four weeks of abstinence experiencing insomnia [11–14]. The most common risk factors of insomnia among AUD patients include more severe AUD, frequent use of alcohol for sleep, concomitant drug use, and psychiatric comorbidities [15, 16]. Additionally, insomnia has been shown to persist for months to years if left untreated in adults with AUD, and, of particular clinical relevance, it has been shown to independently predict alcohol relapse [11, 17–20]. In one seminal study, patients receiving treatment for AUD completed the Sleep Disorders Questionnaire (SDQ) and measures of alcohol use on average 31.5 ± 15.5 days after their last drink [11]. At 5-month follow-up, 48.6% (36/74) had relapsed; among this group, AUD patients classified by the SDQ as having insomnia at baseline were more likely to relapse (59.6%) than those without insomnia (29.6%, *p* = 0.02). After controlling for AUD severity and other covariates, baseline insomnia was the only significant predictor of alcohol relapse [11]. Other studies have failed to identify direct associations between self-reported sleep quality and relapse, but have found that clinical correlates of insomnia (use of alcohol and hypnotics to sleep) significantly predict relapse at 12 months [21]. Consequently, insomnia represents a viable target to supplement existing AUD treatments, improve outcomes, and reduce the burden of illness associated with AUD.

### Treatment of insomnia in AUD

Efforts to target insomnia in patients with AUD are complicated by concerns related to the addiction potential of first-line hypnotic medications and limited evidence in this population for preferred non-medication treatments. Cognitive behavioral therapy (CBT) for insomnia, the most evidence-based non-medication insomnia treatment, is a multicomponent intervention targeting cognitive and behavioral factors that contribute to chronic sleep disturbances. Multiple controlled trials indicate that CBT for insomnia benefits 70-80% of non-AUD individuals with chronic insomnia [22–24], nearly 50% achieve remission from insomnia post-treatment [25, 26], and initial treatment gains are well-maintained over time, with follow-up periods as long as 2-3 years [27–30].

Three randomized controlled pilot trials of CBT for insomnia have been conducted to date in adults with AUD, all of which found robust improvements in sleep and daytime symptoms at post-treatment and follow-up [31–33]. None of these trials found differences in relapse rates; however, two were not based in AUD treatment samples and none of the studies was sufficiently powered to examine alcohol-related outcomes. Moreover, study eligibility for two of the studies required participants to be in early sustained abstinence at entry, as many as 40% of participants reported abstinence > 1 year at baseline, and subjects contracted not to drink during treatment in one of the studies. Consequently, only 15% [31] and 11% [32] of completers in these studies had confirmed relapses, less than half the rate typically found in AUD treatment studies [34], and too low to address the question of whether CBT for insomnia impacts alcohol relapse. Consequently, while insomnia remains a promising target to improve AUD treatment outcomes, the impact of insomnia treatment on alcohol relapse has not been adequately evaluated.

### Mechanisms underlying associations between insomnia and relapse in AUD

The specific mechanisms underlying the association between insomnia and relapse in AUD are poorly understood. Laboratory-based studies using polysomnography indicate pervasive sleep discontinuity (increased sleep latency, decreased total sleep time and sleep efficiency) and altered sleep architecture (reduced slow wave sleep, increased rapid eye movement [REM] sleep) among abstinent adults with AUD, which persist long-term despite continued abstinence [35]. Beyond changes in visually scored sleep architecture, studies also suggest abnormalities in quantifiable sleep mechanisms among abstinent AUD patients.

Sleep is regulated by two fundamentally distinct but interacting systems: a circadian Process C and a homeostatically-driven Process S [36, 37]. Process C reflects the intrinsic biological clock, which promotes wakefulness during the day and regulates the timing of sleep. Process S reflects “sleep drive,” which builds with wakefulness during the day and then dissipates during sleep. Process S is most commonly evaluated by EEG slow wave activity (SWA, EEG frequencies 0.5 to 4.0 Hz) through measurement of SWA power (measured in μV^2^/Hz) and SWA dynamics across non-rapid eye movement (NREM) periods of sleep (measured by exponential regressions). The interaction of Processes C and S determine the daily course of sleepiness, alertness, cognitive performance, and mood [38, 39].

Several studies indicate that Process S, or sleep homeostasis, is severely disrupted in abstinent AUD subjects [40–45]. Compared with matched controls, Colrain and colleagues [45] found subjects with AUD (mean age 49.1 ± 8.5 years, mean 179 days abstinent) had reduced SWA power in the first NREM period and across the entire night. Subjects with AUD in the first month of abstinence also show abnormal responses to sleep deprivation or sleep delay challenges compared to healthy controls, with less SWA power in the first NREM period and slower SWA dissipation across the night [41, 43]. The findings from these studies are consistent with impaired homeostatic sleep regulation; however, no study has evaluated sleep homeostasis in a treatment sample of adults with AUD, associations between this mechanism and clinical outcomes, or whether CBT, which increases SWA in non-AUD insomnia [46], improves sleep homeostasis in patients with AUD and insomnia.

### Impact of CBT for insomnia on sleep homeostasis

Available data, though limited, suggest that CBT may improve homeostatic sleep drive in non-AUD adults with insomnia. One small study of 9 drug-free patients with chronic insomnia (mean age 47 ± 9.7 years, 7 women) found that, relative to baseline, SWA power increased and SWA dissipated at a faster rate after 8 weeks of CBT [47]. In a larger secondary data analysis of a randomized controlled trial, Krystal and colleagues [46] found that CBT (*n* = 16) led to a more rapid dissipation of SWA power across NREM sleep periods compared to a placebo control condition (*n* = 14). Among CBT participants, an increase in peak SWA power in the first NREM period and in the steepness of decline of SWA across NREM periods were positively associated with treatment response. These studies suggest that CBT for insomnia can improve homeostatic sleep drive in adults with insomnia and that improvement in sleep homeostasis is positively associated with treatment response. The current study proposes to extend these findings to a large sample of individuals with AUD and insomnia and to determine the extent to which changes in this sleep mechanism mediate changes in key clinical outcomes (i.e., alcohol relapse).

### Study objectives

The main objectives of this study are (1) to evaluate the benefits of CBT for insomnia on sleep, drinking, and associated daytime symptom outcomes in adults in AUD treatment with insomnia and (2) to determine the association between treatment-related changes in the homeostatic sleep system and relevant clinical outcomes. We will reduce participant burden and enhance innovation and reach by utilizing telemedicine-delivery of both the active (CBT for Insomnia, CBT-TM) and control (Sleep Hygiene Education, SHE-TM) interventions. The study specific aims and associated hypotheses are shown in Table 1. A secondary aim will examine the extent to which changes in alcohol use are mediated by changes in the CBT-TM (vs. SHE-TM) effects on the homeostatic sleep system.

**Table 1:** Study aims and hypotheses

*Aim 1:* To determine whether CBT-TM improves insomnia and daytime symptoms more than SHE-TM in adults in AUD treatment with insomnia.	*Hypothesis 1a*: Insomnia (measured with the Insomnia Severity Index) and fatigue (Multidimensional Fatigue Inventory) will improve more at post-treatment with CBT-TM than SHE-TM. *Hypothesis 1b:* Improvements in insomnia and fatigue will be sustained at 3-, 6-, and 12-month follow-up
*Aim 2:* To compare the efficacy of CBT-TM to SHE-TM on alcohol relapse.	*Hypothesis 2a:* Subjects randomized to CBT-TM will have a higher percentage of days abstinent at post-treatment than those assigned to SHE-TM. *Hypothesis 2b:* This difference will be sustained at 3-, 6-, and 12-month follow-up.
*Aim 3:* To compare the effects of CBT-TM to SHE-TM on homeostatic sleep drive.	*Hypothesis 3:* The rate of SWA dissipation in Non-REM (NREM) sleep (indexed by the exponential regression slope) will increase more from baseline to post-treatment for subjects randomized to CBT-TM than to SHE-TM.

## Methods and design

### Study design and setting

**Fig. 1:**
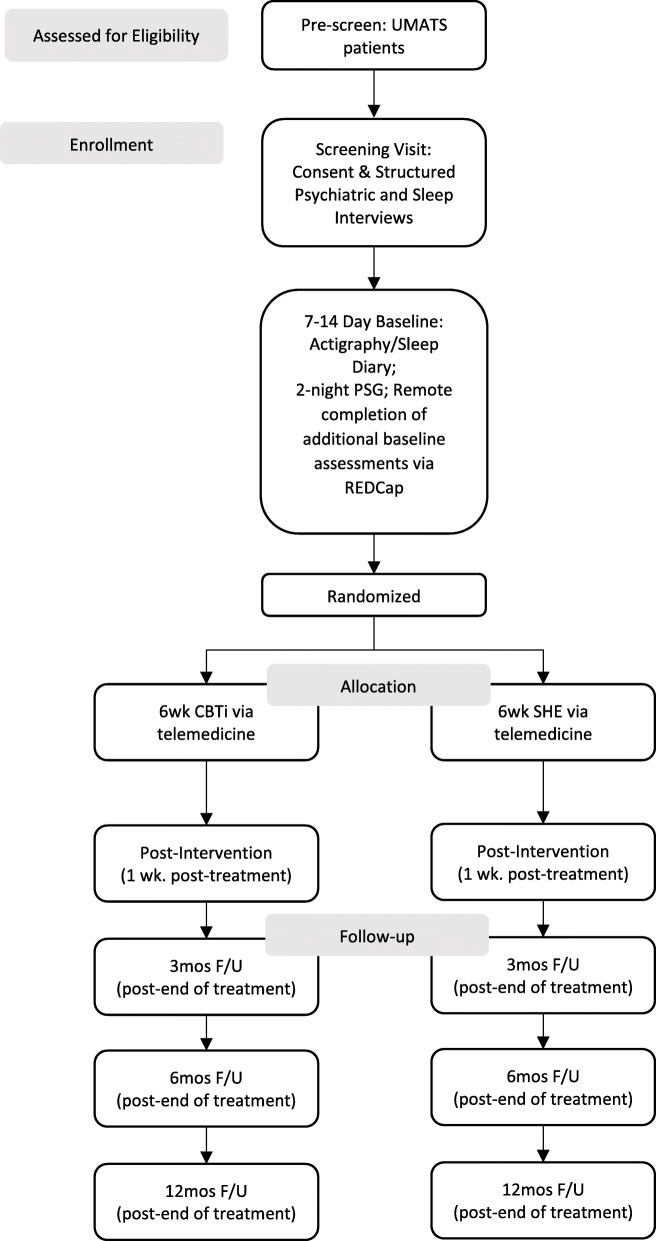
Study flow. *UMATS *University of Michigan Addiction TreatmentServices

This is a randomized controlled clinical trial in which participants (*n* = 150) are randomized to 6 sessions of individual telemedicine-delivered Cognitive Behavioral Therapy for insomnia (CBT-TM, *n* = 75) or Sleep Hygiene Education (SHE-TM, *n* = 75). All participants will complete outcome assessments at baseline, immediately post-treatment, and at 3, 6, and 12 months following completion of treatment. The primary clinical outcomes will be post-treatment scores on the Insomnia Severity Index (ISI), Multidimensional Fatigue Inventory (MFI), and percentage of days abstinent, as determined using a Timeline Follow Back assessment of drinking behaviors. Post-treatment dissipation of SWA in NREM sleep (measured with the best-fit slope based on exponential regressions) will be the primary outcome to assess EEG delta activity, the marker of sleep homeostasis. The study flow is shown in Fig. 1.

The study will be performed at a single site, the University of Michigan, in Ann Arbor, MI. The study protocol has received ethics approval from the Institutional Review Board of the University ofMichigan Medical School (IRBMED) for the University of Michigan and any future amendments to the study protocol will be reviewed by IRBMED.

### Participants

A total of 150 participants will be recruited from patients receiving an episode of abstinence-based outpatient alcohol treatment and from the community via online advertisements (e.g., Facebook, Instagram). Concurrent medications and/or therapies for AUD or co-occurring psychiatric and medical conditions will be permitted. Study participants will meet all of the eligibility criteria outlined in Table 2.

**Table 2:** Participant inclusion and exclusion criteria

Inclusion criteria	- 18–65 years of age at enrollment - Meet DSM-5 criteria for Alcohol Use Disorder (AUD) with ≤ 12 weeks of abstinence - Engaged in an abstinence-based alcohol treatment program - Meet DSM-5 criteria for chronic insomnia, confirmed with sleep diary - Reported stable residence (e.g., reliable place to sleep) - Ability and willingness to travel to Ann Arbor for sleep laboratory assessments or to complete sleep testing at home - For individuals taking medications to promote sleep: agree to maintain a stable regimen from time of enrollment through end of the active treatment phase - Access to Zoom-capable device and Wi-Fi network
Exclusion Criteria	- Diagnosis of, or high suspicion for, sleep disorders other than insomnia - Meet DSM-5 criteria for bipolar disorder, psychotic disorder, or PTSD - Terminal or progressive physical illness (e.g., cancer), neurological degenerative disease (e.g., dementia), or presence of an unstable medical condition that is the specific cause of insomnia - Previous trial of CBT for insomnia - Self-reported pregnancy or intention to become pregnant during the study - Other conditions and situations, medical or otherwise, that preclude meaningful and/or safe participation in CBT/SHE and study procedures

### Measures

#### Screening measures

Participants are screened initially using the Insomnia Severity Index (ISI) and Alcohol Use Disorders Identification Test (AUDIT) to assess for likely chronic insomnia disorder and alcohol use disorder, respectively. For participants recruited in person, these measures are delivered at the initial clinic visit and will be verified via review of the electronic medical record. For participants recruited online, a separate online screening tool includes these measures and is completed to assess eligibility. Additional screening measures include the Mini International Neuropsychiatric Inventory (MINI), to confirm AUD and assess for exclusionary psychiatric conditions, and the Structured Clinical Interview for Sleep Disorders- Revised (SCISD-R) to confirm chronic insomnia diagnosis and rule out other clinical sleep disorders. The final screening assessment is polysomnography (PSG) conducted in-laboratory or at home (using the Prodigy Sleep System, Cerebra Medical, Winnipeg, Manitoba, Canada) to rule out a likely clinical sleep disorder per the International Classification of Sleep Disorders, 3rd Edition (ICSD-3) [48]. Any participant with a PSG suggestive of a clinical sleep disorder other than insomnia will be withdrawn from further study participation and referred for further clinical evaluation and management.

#### Sleep diary

Participants will be asked to complete the consensus daily sleep diary [49] with two additional questions about alcoholic beverage quantity and use of alcohol for sleep at the following time points:

- Baseline period (1–2 weeks before treatment) to confirm insomnia
- Throughout the duration of treatment (6 weeks)
- For 1 week immediately post-treatment and at 3, 6, and 12 months post-treatment

The sleep diary data will be collected daily via a web-based platform. Participants are asked to provide subjective reports of the following: bedtime, lights out time, sleep onset latency, number of nocturnal awakenings, duration of nocturnal awakenings, wake time, and rise time. Additional questions ask about alcohol use in the preceding day and will be used to monitor drinking behaviors and days abstinent.

#### Polysomnography

During the baseline period, participants will complete two nights of in-laboratory or at-home polysomnography (PSG); the first night of data will serve as an adaptation/screening night and the second night will be used as baseline data. Following completion of treatment, participants will be scheduled for an additional night of PSG in the same setting (in-lab or at home) to evaluate objective sleep outcomes.

For in-lab PSG, electroencephalograms (EEGs) will be placed according to the International 10-20 System and recorded from the left and right frontal, central, and occipital electrode sites (F3 & F4; C3 & C4; O1 & O2) [50]; monopolar electooculographic (EOG) recordings will be made from the supraorbital and infraorbital ridges of the eyes; and the electromyogram (EMG) will be recorded from a bipolar chin-cheek montage. Electrophysiological signals will be acquired and analyzed on Compumedics Diagnostic Amplifier Systems using Compumedics Profusion SLEEP4 Online Acquisition and Analysis Software (Compumedics USA Inc., Charlotte, NC). Data will be digitized at 256 Hz and stored off-line for visual stage scoring by a trained sleep technician blinded to participant sociodemographic characteristics and treatment condition [51].

For participants who are unable or unwilling to come for in-lab PSG, especially in consideration of safe public health practices related to COVID-19, PSG will be completed at home using the Prodigy Sleep System device (Cerebra Medical, Winnipeg, Manitoba, Canada). The Prodigy Sleep System is a Class II medical device that has been approved by Health Canada and is currently seeking Food and Drug Administration (FDA) approval in the US. Participants tested with this device will be shipped all of the materials needed to complete testing, full instructions for use, and pre-paid materials to return the device to the study team upon completion. The Prodigy Sleep System utilizes dual frontal EEG leads (F3 & F4 derivations), dual supraorbital/infraorbital EOG leads, intercostal and mental EMG leads, ECG, along with a wireless oximeter, respiratory effort belts, and nasal cannula to collect data comparable to an in-lab PSG. Electrophysiological signal data are sent to a tablet via Bluetooth, and the study team will download data from the tablet to Cerebra Medical’s Michele software, where the raw data will be manually scored by a trained sleep technician blinded to participant sociodemographic characteristics and treatment condition.

#### Actigraphy

Participants are asked to wear an actigraph during the baseline and for 1 week at each follow-up visit (immediately post-treatment, 3, 6, and 12 months post-treatment). The actigraph (Actiwatch Spectrum Plus, Philips Respironics, Bend, OR) is a small waterproof device the size of a wristwatch (48 × 37 × 14 mm weighing 30 g) that is worn continuously on the non-dominant wrist. A solid-state piezo-electric accelerometer (range 0.5–2G peak value, sensitivity 0.025G) integrates movement frequency and intensity into a single measurement. In addition, color-sensitive photodiode sensors (range 400–700 nm) continuously monitor incoming light and store the data in lux levels. The actigraphs are set at a sampling rate of 30 s. Activity and light data are downloaded to computer and sleep/wake activity is estimated by Actiware®–Sleep software using published guidelines [52, 53].

#### Outcome measures

Primary outcomes will be assessed in the following domains:

- *Insomnia (Aim 1)*. Changes in insomnia severity from baseline to post-treatment will be assessed with the Insomnia Severity Index (ISI), a 7-item self-assessment of global insomnia severity that ranges from 0 to 28, with higher scores indicating more severe insomnia symptoms (0–7 no clinical insomnia, 8–14 mild insomnia severity, 15–22 moderate insomnia severity, 23–28 severe insomnia severity). The primary outcome from this measure is post-treatment total score. Secondary outcomes will include post-treatment and follow-up differences in the percentage of responders (ISI change score > 7 relative to baseline) and remitters (post-treatment and follow-up ISI score ≤ 7).
- *Daytime symptoms (Aim 1).* The Multidimensional Fatigue Inventory (MFI) will be the primary measure of daytime symptoms. This 20-item self-report assessment provides an overall fatigue score (20–100) in addition to 5 separate dimensions of fatigue: general fatigue, physical fatigue, reduced motivation, reduced activity, and mental fatigue. Subscale scores range from 4 to 20 with higher scores indicating higher levels of fatigue [54]. The primary outcome from the MFI is the post-treatment general fatigue subscale score.
- *Drinking behavior (Aim 2)*. Changes in drinking behavior will be evaluated with the Alcohol Timeline Follow-Back (TLFB). This interviewer-administered drinking measure assesses both frequency and quantity of alcohol consumption. The primary outcome on the TLFB will be the post-treatment percentage of days abstinent. Secondary TLFB outcomes will include the percentage of heavy drinking days and drinks per drinking day [55].
- *EEG delta activity (Aim 3).* We will use a power spectral analysis (PSA) algorithm to evaluate EEG delta activity. PSA generates power (area under the curve) in five EEG frequency bands: delta (0.5 to < 4 Hz), theta (4 to < 8 Hz), alpha (8 to < 12 Hz), sigma (12 to < 16 Hz), beta (16–32 Hz), and gamma (> 32 Hz), expressed in μV2. Power values are averaged across 30s epochs for each frequency band, generating data segments in identical epoch lengths to the stage-scored data [56]. Power values are then sorted by sleep stage to evaluate the distribution of EEG frequencies within and across successive REM and NREM periods [57, 58]. The primary outcome will be the post-treatment dissipation of SWA in NREM sleep (measured with the best-fit slope based on exponential regressions), but we will additionally evaluate SWA power in the first NREM period and averaged across the night.

To evaluate treatment-related changes in daytime functioning, we will also administer the following secondary outcome measures (see Table 3 for schedule of assessments):

- *Morningness Eveningness Questionnaire (MEQ)*: a 19-item scale to assess participant-preferred tendencies for timing of daytime/nighttime activities and assessment of different circadian rhythm chronotypes.
- *Epworth Sleepiness Scale (ESS)*: an 8-item scale used to assess for the severity of daytime sleepiness.
- *Dysfunctional Beliefs and Attitudes About Sleep (DBAS)*: a 16-item scale used to identify unhelpful beliefs about sleep and insomnia.
- *Generalized Anxiety Disorder (GAD-7)*: a 7-item scale to assess for generalized anxiety disorder symptoms.
- *Patient Health Questionnaire Depression Scale (PHQ-8)*: an 8-item scale designed to screen for symptoms of depression. This version does not include the item to assess for suicidal ideation, which is assessed elsewhere (MINI).
- *12-item Short Form Survey (SF-12)*: a 12-item scale to assess participants’ perception of overall health and well-being.
- *Short Inventory of Problems-Revised (SIP-R)*: a 17-item scale to examine the adverse consequences of drug and/or alcohol abuse.
- *Penn Alcohol Craving Scale (PACS)*: a 5-item scale to examine frequency, duration, and intensity of alcohol cravings.

**Table 3:** Timing of assessments and interventions throughout study enrollment

	Pre-Randomization			Intervention (6 sessions)						Post^4^	3m FU^4^	6m FU^4^	12m FU^4^
	SCRN^1^	BSL^2^		Wk3	Wk4	Wk5	Wk6	Wk7	Wk8	Wk9	Wk20	Wk32	Wk60
	V1	V2	V3^3^	S1	S2	S3	S4	S5	S6	V4^3^	V5	V6	V7
Consent		X											
Psychiatric Screening	X												
Demographics	X												
AUDIT	X												X
Structured psychiatric interview		X											
Structured sleep interview		X											
PSG			X							X			
Actigraphy			X							X	X	X	X
Sleep diary			X	X	X	X	X	X	X	X	X	X	X
ISI	X		X							X	X	X	X
MEQ			X							X			
ESS			X							X	X	X	X
DBAS			X							X			
MFI-20			X							X	X	X	X
GAD-7			X							X	X	X	X
PHQ-8			X							X	X	X	X
SF-12			X							X	X	X	X
SIP-R			X							X	X	X	X
PACS			X							X	X	X	X
TLFB			X							X	X	X	X
CBT-TM or SHE-TM				X	X	X	X	X	X				
TEQ				X						X			
WAI				X		X				X			
Comprehension				X						X			

(1) At screening, ISI and AUDIT eligibility will be confirmed via chart review or online questionnaire. Demographics and psychiatric screening will come from a combination of chart review and prospective participant questionnaire. (2) Baseline assessments will be collected as a mixture of in-person and remote interactions. (3) V3 represents the period between V2 and S1. V3 and V4 will last approximately 7–14 days, during which time participants will undergo PSG and maintain sleep diary/actigraphy. Participants will complete baseline assessments via REDCap only after passing baseline PSG. (4) All post-intervention visits are determined relative to treatment completion date

Finally, the following treatment process measures will be administered:

- *Therapist Evaluation Questionnaire (TEQ)*: 5-item self-report scale that assesses perceived logic, confidence, and success of the treatment and participant willingness to take part.
- *Working Alliance Inventory – Short Revised (WAI-SR)*: a 12-item measure that evaluates three aspects of therapeutic alliance—agreement on therapy goals and tasks, and development of an affective bond.

### Procedures

Participants are recruited either from abstinence-based outpatient alcohol treatment programs or from the community. All participants will be scheduled for a combined consent and baseline visit, to be conducted remotely, via BlueJeans (BlueJeans Network, San Jose, CA) or Zoom for Healthcare (Zoom Video Communications, Inc., San Jose, CA), or in person, as conditions permit. Consent for audio recording of treatment sessions for treatment fidelity review, submission of data to the NIAAA Data Archive, and storage of de-identified data for unspecified future research will also be sought at this time. After the participant and team member have signed the ICF, the study team member will deliver the MINI and SCISD-R screening assessments to assess participant eligibility. After screening assessments confirm diagnoses of insomnia and alcohol use disorder, and rule out any exclusionary criteria, participants will be provided with a daily sleep diary and actigraph to maintain during the baseline period to confirm insomnia. They will also be scheduled for either in-lab or at-home PSG at this time. Following completion of all baseline eligibility measures, randomization will be completed according to the procedures outlined below (see the “Randomization and blinding” section).

Follow up assessment begins immediately after the completion of treatment when participants complete post-treatment assessments, one additional night of PSG, and 1 week of sleep diary/actigraphy. At 3, 6, and 12 months post-treatment, participants will again complete self-report assessments and 1 week of sleep diary/actigraphy.

Once enrolled, we will utilize a variety of retention strategies that have been successful in our previous studies. These strategies include (1) requesting participants to designate at least two contact persons to assist in locating them as needed, (2) providing participants with multiple reminder phone calls before appointments, (3) sending follow-up letters by mail to maintain participant engagement, (4) encouraging continuity of contact with individuals on the research team (participants will be remunerated with $5 at each follow-up time point for contacting the research team to schedule follow-up assessments and update contact information), (5) offering participants financial compensation for completion of study activities, and (6) an analytic approach that minimizes the impact of missing data on estimation of outcomes.

Throughout participation, any adverse events (AEs), both solicited and spontaneously reported, will be reported to the Principal Investigator, who has ultimate responsibility to determine severity and relatedness of any events. Events will then be reported to IRBMED and NIH, following reporting guidelines based on severity and relatedness of AEs.

#### Randomization and blinding

Randomization will be performed with a centralized computerized application, Treatment Assignment Tool - University of Michigan (TATUM), with random block sizes of up to 10, and stratified based on participant gender, age (≤40 or > 40 years old), and presence vs. absence of a substance use disorder. Randomization is performed the study coordinator, who will enter necessary data for stratification into the system, which will then return a treatment assignment. Allocation to treatment condition by the study team member is conducted only after all study eligibility criteria have been met. Randomization will allocate participants in a 1:1 ratio to 6 sessions of either CBT-TM or SHE-TM.

Subjects will not be informed of the treatment assignment in order to prevent bias in response. Additionally, research staff assessing outcomes, including research assistants, data analysts, and polysomnography technicians, will be blinded to treatment condition to help ensure a rigorous and unbiased approach. The study coordinator has the responsibility to complete allocation to treatment groups, as outlined above, and thus cannot be blinded to treatment condition. Therapists are blinded to study outcomes but are not blinded to treatment condition (CBT-TM vs. SHE-TM); they will use rigorous treatment fidelity procedures (see below) to minimize the risk of bias in our findings. Data will be coded to preserve blinding for treatment sessions, such that any blinded research staff cannot determine the treatment assignment of any participant.

### Interventions

Participants will be randomized to complete 6 sessions of telemedicine-delivered Cognitive Behavioral Therapy for Insomnia (CBT-TM) or Sleep Hygiene Education (SHE-TM). Both interventions will be delivered remotely by a Master’s-level therapist familiar with time-limited and evidence-based therapies who has been trained on the study interventions by a study investigator with a Diplomate in Behavioral Sleep Medicine (DAC). Prior to delivering therapy, the therapist will demonstrate understanding of the material by successfully completing a written examination. Both interventions are designed to be delivered over 6 consecutive weeks and each session lasts between 30 and 60 min, with different topics covered each week, as outlined in Table 4.

**Table 4:** Session-by-session breakdown of study interventions

*Telemedicine-delivered Cognitive Behavioral Therapy for Insomnia (CBT-TM)*		
Session 1	45–60 min	**CBT-TM Introduction/Sleep Hygiene—**orientation to insomnia and CBT. Identify and alter daytime behaviors, substances, and environmental conditions that can disrupt sleep
Session 2	45–60 min	**Sleep Scheduling Strategies (Sleep Restriction/Stimulus Control)—**improves sleep quality, duration, and timing with behavioral strategies that increase the drive for sleep and stabilize the circadian timing system.
Session 3	60 min	**Counter-Arousal Strategies—**reduces physiological and cognitive arousal through constructive worry and relaxation. Time in bed adjustments will be made according to an algorithm that combines sleep diary information with patient-reported side effects during the day
Session 4	30–45 min	**Cognitive Therapy—**aims to identify and alter dysfunctional beliefs about sleep and functioning that contribute to maintaining insomnia. Time in bed adjustments continue.
Session 5	30–45 min	
Session 6	45–60 min	**Relapse Prevention for Insomnia—**reviews treatment gains, emphasizing the behaviors and adaptive cognitions necessary for maintaining these gains. Relapse prevention of insomnia involves identifying high-risk situations, promoting realistic appraisals about future insomnia episodes, and providing behavioral and cognitive strategies for dealing with the inevitable occasional poor night of sleep [59].
*Telemedicine-delivered Sleep Hygiene Education (SHE-TM)*		
Session 1	45–60 min	**Insomnia History—**reviews the participant’s insomnia history, including triggers that initiated the sleep problem, its duration, severity, and frequency, premorbid sleep characteristics, and previous sleep medication and non-medication treatments.
Session 2	45–60 min	**Sleep Education—**provides basic education about why we sleep, sleep stages, how sleep is regulated at night, and sleep changes across the lifespan
Session 3	45–60 min	**Substance Use and Sleep—**focuses on the effects of alcohol and other licit and illicit substances on sleep
Session 4	30–45 min	**Environmental Factors and Sleep—**focuses on the importance of creating a sleep-conducive environment for optimizing sleep quality
Session 5	30–45 min	**Lifestyle factors affecting Sleep—**addresses the effects of diet, exercise, and napping on sleep. Subjects will also be educated about the importance of regularity of activity and meal times to enhance sleep quality
Session 6	45–60 min	**Sleep Maintenance Strategies—**reviews treatment gains and emphasizes the principles covered over the previous five sessions in order to maintain sleep improvements

#### Treatment monitoring and therapist fidelity

Participant treatment fidelity will be assessed by tracking session attendance and reschedules/cancellations. Additionally, participants will be given a content comprehension measure following the first therapy session and at the final treatment visit.

A random sample of 25% of treatment session videos will be reviewed by the study therapist supervisor (DAC). Each of these videos will be scored according to a Treatment Fidelity and Therapist Competence measure designed for this study, which include monitoring to ensure that critical session topics are covered and that the therapist demonstrates competence in basic therapy skills. In addition, the therapist will be assessed by the participant’s completion of the Working Alliance Inventory and Therapist Evaluation Questionnaire.

Participant adherence with treatment protocols will be monitored by the study therapists via daily sleep diaries. Treatment will be modified or discontinued if adverse events are reported. During the trial, any necessary treatment for co-occurring medical, mental health, or alcohol/substance use disorder conditions is permitted.

### Data management and analysis

#### Data collection and management

The Project Coordinator and Data Manager will directly oversee the day-to-day data collection and quality assurance activities. Participant surveys will be entered directly into a REDCap database by participants, with monthly checks by a study team member for completeness, accuracy, and out-of-range values. Built-in and study-specific data validation rules in our study database will be used to facilitate these checks. Actigraphy and PSG data will be stored in separate proprietary databases associated with the systems being used and will be stored without any identifiable information. Routine data backups will be part of this process. Collection of identifiable data from participants will be kept to the minimum necessary to carry out study procedures; this data will be kept in a secured, password-protected REDCap database. There will not be any biological specimens collected or analyzed for this study.

De-identified trial data will be uploaded into the NIAAA Data Archive (NIAAA_DA_) biannually, making it accessible to other researchers interested in the treatment of sleep disorders and/or alcohol use disorder. Trial results will be communicated via presentations at national scientific meetings, publication in peer-reviewed journals, and dissemination of aggregate data to local clinics.

#### Data and safety monitoring plan

A Data and Safety Monitoring Board (DSMB) will be appointed to review study data and monitor participant safety on an annual basis. This committee will be comprised of experts in the treatment of AUD, sleep medicine, and statistics, respectively; DSMB members are independent of the study team and of the study sponsor, the NIAAA. The DSMB will meet annually to review study progress and data to determine whether interim data analyses are required and will have ultimate responsibility for determining whether any intercurrent safety considerations require amendments to this study protocol or study termination. Additionally, the DSMB is responsible for making recommendations regarding the efficacy of the intervention, risk/benefit ratios, issues of participant burden, and other concerns as the DSMB deems relevant. There is no anticipated harm to participants from study participation and, as such, no provisions for post-trial or ancillary care have been made.

A study team member will review consent forms, case report forms (CRF), and source data 2 weeks after the first subject is consented for screening, 2 weeks after the first subject is randomized, and then on at least a quarterly basis. Each review will be documented in the Monitoring Log and accompanied by a written Monitoring Report to be filed in the Regulatory Binder.

#### Statistical considerations

We will utilize an “intent-to-treat” (ITT) approach, meaning that all randomized subjects will be included in analyses, regardless of whether they complete all assessments, and all hypothesis testing will use two-sided tests (*p*-values < 0.05 will be considered significant). Analyses will begin with descriptive examinations of distributions of key variables collapsed by categories, if necessary, and to review data for outliers and clear anomalies. Tests for linearity, independence, missingness, and distributional assumptions will be performed. Normalizing transformations will be utilized, if necessary. Prior to fitting any analytic models, a graphical exploration of the outcome variables will be conducted. Primary analyses will focus on the overall sample. We will validate randomization by testing for covariate balance between groups. If differences are observed, we will adjust for these variables or conduct stratified analyses.

To minimize missing data, we will make every effort to gather follow-up information from all participants using strategies that have proved successful in our prior research. Where we have missing variables that are necessary for analysis, we will perform multiple imputation of missing data and combine the results from 5 imputations based on Rubin’s method to produce an estimate and the corresponding confidence interval accounting for missing data uncertainty. It is important to note that the proposed mixed-effects regression modeling will allow for the use of data from all participants and provide unbiased parameter estimates that account for missing data under the missing-at-random assumption. We will also examine for non-random attrition, and if we find, for example, attrition rates to depend on a baseline covariate, we will include this covariate in the modeling.

Bivariate associations between independent variables (including treatment group) and primary outcomes will be examined prior to model building. For example, we will examine correlations and compare groups using appropriate comparison methods such as *t*-tests, *F*-tests, and bivariate generalized linear models with specification of the appropriate link function based on the distribution of the given outcome, or their non-parametric counterparts (e.g., Mann-Whitney). To compare intervention conditions, we will also compare average pre-post differences on the outcomes of interest.

Generalized linear mixed models (GLMMs) will be the focal models used to examine treatment effects and changes in the primary outcome measures of interest. We chose GLMMs because (1) GLMMs take into account correlations between data points (e.g., repeated measurements on individuals) and (2) GLMMs allow for the retention of all participants in the analysis, including those with incomplete data. Primary outcome variables include those with Gaussian distributions (e.g., insomnia severity), as well as those with count processes (e.g., percent days abstinent) that likely follow a Poisson distribution. Nonetheless, such assumptions will be scrutinized and, as needed, modifications (e.g., zero-inflation) and alternative families of distributions (e.g., negative binomial) will be considered. An autoregressive covariance specification will be used first for GLMMs, but appropriateness of other covariance specification will also be considered.

#### Primary outcome analyses

##### Specific aim 1: Between group treatment effects on insomnia and daytime symptoms

We will assess treatment effects on insomnia and daytime symptom outcomes at post-treatment and at 3-, 6-, and 12-month follow-up via bivariate analyses described above, comparing the CBT-TM to SHE-TM. We will then estimate treatment effects on insomnia and fatigue using a GLMM approach. An indicator variable for the CBT-TM group will be used to generate estimates in relation to SHE-TM. If preliminary analyses suggest that the effect of the intervention may vary over time, we will model an interaction of intervention by time. In addition to the treatment group indicator, GLMMs will include the baseline values of the response variable as an independent variable.

- Primary outcomes: Post-treatment ISI total score and MFI General Fatigue subscale score
- Exploratory outcomes: Other measures of insomnia and daytime symptoms as captured by self-report measures, sleep diary, and actigraphy measures across time points

##### Specific aim 2: Between group efficacy for alcohol relapse

We expect that the primary drinking outcome (percent days abstinent) will likely follow a Poisson distribution, so GLMM models will be specified accordingly (e.g., with specification of the log link function). Nonetheless, as described above, assumptions will be scrutinized and adjusted, if necessary. Analyses will then proceed as described above for Aim 1 assessing treatment effects on drinking outcomes.

- Primary outcome: percent days abstinent as measured by TLFB at post-treatment
- Exploratory outcomes: Other indices of drinking-related behavior including percentage of heavy drinking days and drinks/drinking day, etc. as measured by TLFB across time points

##### Specific aim 3: Treatment effects on homeostatic sleep drive

The analytic procedures for Aim 3 will be similar to those described by Krystal and Edinger [46]. We will compare changes between baseline and post-treatment SWA outcomes for the CBT-TM and SHE-TM groups. The primary outcome will be post-treatment dissipation of SWA in NREM sleep (i.e., best-fit slope of delta power, where the slope is the exponent of best-fit exponential function). To assess for differences in change of slopes between the conditions, we will use analysis of variance (ANOVA) modeling. We chose ANOVA modeling to be consistent with the prior work and because GLMMs offer little advantage over ANOVAs with only two time points. Similar modeling procedures will be used to assess differences for secondary Aim 3 outcomes (e.g., baseline-to-post-treatment change in SWA power in the first NREM period averaged over the entire night) assessed during PSG.

#### Sample size and detectable effect size

Target sample size for this study is 150 participants, with allocation of 75 participants to each treatment group. Power analyses were conducted using G*Power 3.1.9 software, and assumed a two-sided test with *α* = .05. Because of the lack of commercially available software for calculating power for GLMMs, we have conservatively estimated power for traditional regression approaches for the specific aims without accounting for GLMM methods that will allow for inclusion of participants with partial follow-up data or for the gains in power generated by the use of repeated measures. Power was based on a conservative estimate of *n* = 120 individuals (*n* = 60 in CBT-TM; *n* = 60 in SHE-TM), which represents an 80% follow up rate of the initial sample of *n* = 150, and does not take into account multiple imputation and other strategies for handling missing data.

Based on this conservative approach, we are powered for conducting bivariate comparisons, pre-post testing of intervention effects, and comparing intervention groups and we show the viability of our sample size for detecting realistic effect sizes in the proposed GLMM models. For comparisons between the treatment group (*n* = 60) and the control group (*n* = 60), we have > 80% power to detect a Cohen’s *d* > 0.51 (e.g., differences between groups at a single follow-up), which represents a medium effect size. This effect size, as well as the proposed sample size, is similar to prior published studies of interventions targeting a comorbid condition to reduce alcohol use (e.g., pain or depression interventions aimed at reducing drinking [60, 61]). Consequently, we will have more than adequate power to detect meaningful effect sizes that we expect based on our prior studies of CBT and the extant literature.

## Discussion

Alcohol use disorder (AUD) is a leading preventable cause of morbidity and mortality in the US, with relapse rates remaining unacceptably high following existing treatments. Consequently, novel approaches that address predictors of relapse are critically needed. Insomnia is one such target that could enhance treatments for AUD, improve treatment outcomes, and reduce burden associated with AUD. Our study will be the first adequately powered controlled trial to determine whether CBT for insomnia improves sleep, daytime functioning, and most importantly, key alcohol-related outcomes in treatment-seeking AUD patients. The study extends the current literature by additionally proposing to evaluate treatment-related changes in a fundamental sleep mechanism - sleep homeostasis - and its association with drinking outcomes. Support for the study hypotheses would provide a novel clinical approach to augmenting existing AUD treatments, clarify the impact of CBT for insomnia on sleep homeostasis for adults with AUD, and further understanding of the association between this mechanism of sleep dysfunction and clinical outcomes.

There are several aspects of this study that make it novel. First, the study interventions will be delivered by telemedicine to reduce barriers to participation. We recently published findings indicating that outcomes obtained from telemedicine-delivered CBT for insomnia were not inferior to gold standard face-to-face delivery in a non-AUD sample [62], supporting use of this specific delivery modality. The use of telemedicine delivery will also allow for broader dissemination of this intervention should the study hypotheses be supported. Second, the study focuses specifically on patients in AUD treatment to evaluate whether or not the addition of an insomnia treatment *augments* clinical outcomes for these individuals. Although our primary clinical target is drinking behavior, the intervention may have broader impacts on daytime functioning and quality of life for this patient population. Thus, the findings from this trial have important implications for future clinical care. Third, the project is designed to evaluate the impact of the intervention on a key sleep regulatory mechanism shown to be dysfunctional in individuals with AUD (homeostatic sleep drive) using state-of-the-art laboratory techniques in sleep research. Our group has particular experience in quantitative EEG analyses, permitting us to evaluate changes in EEG SWA following active and control treatments. Finally, the post-treatment follow-up assessments at 3, 6, and 12 months allow for evaluation of the sustained benefits of CBT-TM on both alcohol- and non-alcohol-related clinical outcomes.

This trial was originally proposed to include only in-lab polysomnography and a combination of in-person and remote assessment collection. In light of the COVID-19 pandemic and local restrictions on access to laboratory space and in-person appointments, the protocol has been amended to follow safety recommendations set forth by the state of Michigan and University of Michigan. The changes included the addition of a home sleep test system to conduct sleep study assessments (Prodigy Sleep System, Cerebra Medical, Winnipeg, MB, Canada) and transition to all remote follow-up visits and assessments. As the circumstances surrounding COVID-19 continue to evolve, the study team will closely monitor guidelines from the Centers for Disease Control and local governing bodies in order to responsibly transition to in-lab PSG and in-person study visits.

In summary, this trial is designed to evaluate a novel approach to improve drinking outcomes among patients in AUD treatment with insomnia and to determine if a non-medication, telemedicine-delivered treatment for insomnia improves an identified mechanism of sleep dysfunction in this population. As such, the findings from this study will inform future clinical practice for treatment-seeking adults with AUD and provide scientific insights on the associations between brain mechanisms of sleep and clinical outcomes in this population.

### Trial status

This protocol is Version 1, last updated on 17 June 2020. The trial began participant recruitment on 1 October 2020, with an anticipated completion date of 1 June 2023.

## Data Availability

All data will be submitted to the National Institute on Alcohol Abuse and Alcoholism Data Archive (NIAAADA). All investigators will have access to the finalized dataset.

## Abbreviations

AUD: Alcohol use disorder
CBT-TM: Cognitive Behavioral Therapy for Insomnia, telemedicine
SHE-TM: Sleep Hygiene Education, telemedicine
PSG: Polysomnography
AUDIT: Alcohol Use Disorders Identification Test
ISI: Insomnia Severity Index
TLFB: Timeline Follow-Back Assessment
MFI: Multidimensional Fatigue Inventory
ESS: Epworth Sleepiness Scale
DBAS: Dysfunctional Beliefs and Attitudes About Sleep Scale
TEQ: Therapist Evaluation Questionnaire
WAI: Working Alliance Inventory
MEQ: Morningness Eveningness Questionnaire
EEG: Electroencephalogram
SWA: Slow wave activity
REM: Rapid eye movement
NREM: Non-rapid eye movement
IRBMED: Institutional Review Board of the University ofMichigan Medical School
MINI: Mini International Neuropsychiatric Inventory
SCISD-R: Structured Clinical Interview for Sleep Disorders-Revised
EMG: Electromyogram
ECG: Electrocardiogram
EOG: Electrooculogram
GAD-7: Generalized Anxiety Disorders – 7 scale
PHQ-8: Patient Health Questionnaire
PACS: Penn Alcohol Craving Scale
SIP-R: Short Inventory of Problems-Revised
SF-12: 12-item Short Form Survey
ICF: Informed consent form
OSA: Obstructive sleep apnea
